# Helicobacter pylori and gastroesophageal reflux disease

**DOI:** 10.1186/1477-7819-6-74

**Published:** 2008-07-05

**Authors:** Michele Grande, Federica Cadeddu, Massimo Villa, Grazia Maria Attinà, Marco Gallinella Muzi, Casimiro Nigro, Francesco Rulli, Attilio M Farinon

**Affiliations:** 1 Department of Surgery, University Hospital Tor Vergata, Rome, Italy

## Abstract

**Background:**

The nature of the relationship between *Helicobacter pylori *and reflux oesophagitis is still not clear. To investigate the correlation between Helicobacter pylori infection and GERD taking into account endoscopic, pH-metric and histopathological data.

**Methods:**

Between January 2001 and January 2003 a prospective study was performed in 146 patients with GERD in order to determine the prevalence of Helicobacter pylori infection at gastric mucosa; further the value of the De Meester score endoscopic, manometric and pH-metric parameters, i.e. reflux episodes, pathological reflux episodes and extent of oesophageal acid exposure, of the patients with and without *Helicobacter pylori *infection were studied and statistically compared. Finally, univariate analysis of the above mentioned data were performed in order to evaluate the statistical correlation with reflux esophagitis.

**Results:**

There were no statistically significant differences between the two groups, HP infected and HP negative patients, regarding age, gender and type of symptoms. There was no statistical difference between the two groups regarding severity of symptoms and manometric parameters. The value of the De Meester score and the ph-metric parameters were similar in both groups. On univariate analysis, we observed that hiatal hernia (p = 0,01), LES size (p = 0,05), oesophageal wave length (p = 0,01) and pathological reflux number (p = 0,05) were significantly related to the presence of reflux oesophagitis.

**Conclusion:**

Based on these findings, it seems that there is no significant evidence for an important role for *H. pylori *infection in the development of GERD and erosive esophagitis. Nevertheless, current data do not provide sufficient evidence to define the relationship between HP and GERD. Further assessments in prospective large studies are warranted.

## Background

Helicobacter pylori (HP) has been demonstrated the causative factor of various gastrointestinal diseases; nevertheless, the relationship between HP infection and gastroesophageal reflux disease (GERD) is still debated [1]. To date, different studies have examined the relationship between atrophic gastritis due to HP infection and reflux oesophagitis with conflicting results.

Recent trials suggest that HP infection may be an important causative factor of atrophic gastritis [2]. HP infection has been associated to inflammation of gastric mucosa that increases cellular apoptosis and epithelium proliferation. The excessive apoptosis, leads to the atrophy of epithelial cells and glands and could contribute to carcinogenesis.

Some authors have found an increase of reflux oesophagitis after HP eradication. On the contrary, other authors suggested a correlation between HP infection and presence and severity of reflux esophagitis [3].

It was suggested that HP could contribute to GERD through different mechanisms: cardias inflammation causing sphincter weakness; increased acid secretion due to antral gastritis; delayed gastric emptying and citotoxin production causing esophageal epithelium injury.

Conversely, other authors believe that HP infection may even protect against GERD and HP eradication may lead to an accelerated development of GERD in ulcer disease patients [1,2,4-6]. Further, previous studies have shown an increased effect of proton pump inhibitors on intragastric pH in HP-infected patients suffering from GERD with rapid heartburn relief and lack of relapse [7].

HP could play a protective role through different mechanisms: decrease of acid secretion resulting from chronic gastritis of the gastric body; improvement of gastro-oesophageal junction due to proximal gastritis and finally production of ammonium by the gastric colonization of HP that could be a potential stopgap system [1-10].

The present prospective study was performed in 146 patients with GERD in order to determine the prevalence of Helicobacter pylori (HP) infection at gastric mucosa; furthermore the correlation between HP infection and endoscopic, manometric, pH-metric and histological findings was studied through the statistical comparison of endoscopic, functional and histological data between subjects with and without HP infection. Finally, we analysed the statistical correlation between reflux esophagitis and HP infection, endoscopic, manometric, pH-metric data.

## Materials and methods

Between January 2001 and January 2003, 146 consecutive patients with daily reflux symptoms for at least one year were evaluated at the Department of Surgery, Tor Vergata University Hospital, Rome and were included in this prospective study.

The study had been approved by the Institutional Committee of the Tor Vergata University of Rome.

Exclusion criteria were the following: 1. Previous therapy to eradicate HP. 2. Concomitant assumption of aspirin and non-steroidal anti-inflammatory drugs 3. Previous surgical procedures on digestive tract.

All patients underwent a pre-treatment evaluation, which included anamnesis, clinical examination, EGDS with biopsy, oesophageal manometry and 24 hours pH-metry.

Symptoms (heartburn, pain, and regurgitation) were assessed by patients' visits.

Ambulatory manometry and pH studies were performed using a conventional protocol. A catheter with three pressure sensors (intersensor distance 5 cm) and one pH sensor was used. The catheter was connected to an 8 Mb data-logger with a sampling frequency of 4 Hz. After an overnight fast the catheter was introduced transnasally and placed in the esophagus. The lowermost pressure transducer was placed 2 cm above and the pH sensor placed 5 cm above the upper border of the lower esophageal sphincter. The lower esophageal sphincter was identified by the stepwise pull-through technique.

The pH and motility data were analyzed with the help of a computer program (Multigram, V 6.30, Synectics Medical). The analyses of both pressure and pH data were done separately for the total, upright (upright period excluding the meal period), meal and supine periods according to standard protocols.

Oesophageal manometry was performed in order to define position, extension, pressure of LES (LES pressure: normal range 14,3–34,5 mmHg), esophageal wave length and height (table 1). Oesophageal motility and gastroesophageal junction coordination were evaluated using damp deglutitions of 5 ml water bolus.

**Table 1:** Definition of Esophageal Motility Disorders

**Diagnoses**	**Manometric impairments**
Aperistalsis	
	Absent or simultaneous contractions (<30 mmHg)
	Ineffective esophageal motility
	≥3 peristaltic contractions with failure of wave progression or failed peristalsis over a segment of the distal esophagus
Normal	
	Normal velocity
	Normal peristaltic amplitude
	≥7 peristaltic contractions with an intact wave progression (amplitude >30 mmHg)
Nutcracker esophagus	
	Average peristaltic amplitude >180 mmHg over pressure sensors 3 and 8 cm above LES
Isolated hypertensive LES	
	Basal LES pressure greater than 45 mmHg
Distal esophageal spasm (DES)	
	Contractile velocity >8 cm/s mmHg over pressure sensors 3 and 8 cm above LES in ≥ 2 swallows
Atypical disorders of LES relaxation	
	Abnormal LES relaxation, may have simultaneous or absent peristalsis
Achalasia	
	Abnormal LES relaxation
	Absent or simultaneous contractions

Modified from Pandolfino et al [48]

Twenty-four hours pH-metry was performed taking into account the following parameters: 1. DeMeester score value (normal value up to 14.7); 2. total number of reflux episodes, number of pathological reflux episodes (refluxes with pH<4 that least over 5 minutes); 3. extent of oesophageal acid exposure; 4. type of reflux (in orto- and clinostatism or total); 5. number of long acid reflux episodes 6. extent of the longest pathological reflux.

At endoscopy LES opening, presence of hiatus ernia, evident refluxes and esophagitis were evaluated. Esophagitis was graded by endoscopy according to the Savary-Miller classification: grade 0 indicates no lesions; grade 1, erythema of the mucosa with multiple erythematous and exudative lesions; grade 2, multiple erosions affecting multiple folds, not confluent; grade 3, multiple linear or circumferential erosions that may be confluent; grade 4, ulcer, stricture, or esophageal shortening, Barrett's epithelium.

Barrett's esophagus has been defined as the presence of squamo-columnar metaplasia localized at least 3 cm above the oesophagus-gastric junction; 2–3 samples of the lower oesophagus (last 3 cm) were obtained.

Endoscopic biopsy both of the gastric body and of the antrum was performed in order to diagnose HP infection and to obtain istological evaluation of the mucosa. HP infection was diagnosed by either endoscopic evaluation and color coded biopsy test.

### Statistical analysis

All statistical elaborations were obtained by using Statigraphies 5 plus for Window XP (Statsoft; Tulsa, Okla, USA). Results are expressed as mean values and standard deviation (SD).

Quantitative variables between the two groups (HP positive and HP negative patients) were compared using the Student's t-test; qualitative parameters were compared between the two groups using chi-squared test.

Results were considered statistically significant at *P *< 0.05.

## Results

The present study included 146 patients, 58 males and 88 females with a mean age of 51,5 ± 15,2 years (range 23–89). All patients suffered from daily reflux symptoms for at least one year. HP infection was diagnosed in 35 patients (24%), 13 males and in 22 females, while 111 patients (76%), 45 males and 66 females, were HP negative.

Patients with and without HP infection were statistically compared. There were no significant differences between the two groups regarding age, gender and presentation symptoms.

Hiatal hernia was found in 97 cases out of 146 patients (66.4%); 25 patients were HP positive (25.7%) and 72 were HP negative (74.3%).

Reflux esophagitis was evidenced by endoscopy in 41 patients (28%); according the Savary-Miller classification, out of 146 patients, 105 were graded 0; 14 patients were graded 1–3 (3 HP positive patients and 11 HP negative) and finally 27 patients were graded 4 (9 HP positive patients and 18 HP negative).

Impairment of oesophageal motility was detected at manometry in 111 patients out of 146 (76%); HP was present in 26 of these (23.5%) while 85 were HP negative (76.5%).

There was no statistical difference regarding LES pressure between patients HP positive and HP negative (19,4 ± 95,0 (range 3,7–46.2) and 19,7 ± 115,0 (range 2,6–61) respectively). Further, significant difference was evidenced neither in oesophageal wave length (mean value 3.1 seconds in HP-negative patients vs 3,2 seconds in HP positive) nor in oesophageal wave height (mean value 72,4 ± 39,3 in HP-negative patients vs 67,9 ± 28,4 in HP positive) (table 2).

**Table 2:** Manometric data of 146 GERD patients

**MANOMETRIC PARAMETERS**	**HP positive**	**HP negative**	**P**
**Patients**	35	111	
**LES pressure (mmHg)**	19.4 ± 9.7	19.7 ± 10.7	NS
**Motility impairment**	26 (74.3%)	88 (79.3%)	NS
**Esophageal wave length (sec)**	3.2	3.1	NS
**Esophageal wave height**	67.9 ± 28.	72.4 ± 39.3	NS

The pH-metric parameters, i.e. reflux episodes, pathological reflux episodes and extent of esophageal acid exposure, were similar in both groups (table 3).

**Table 3:** pH-metric data of 146 GERD patients

**pH-METRIC PARAMETERS**	**HP positive**	**HP negative**	**P**
**Patients**	35	111	
**Reflux episodes**	113.9 ± 147.8	135.1 ± 129	NS
**Pathological reflux episodes**	3.3 ± 6.7	2.4 ± 4.3	NS
**Esophageal acid exposure (min)**	117 ± 195.2	109.3 ± 205.5	NS
**De Meester score**	35.9 ± 56.7	33.3 ± 48.4	NS

Out of 146 patients, 75 (51.4%) had pathological values of De Meester score; 17 patients were HP positive (22.7%) and 58 were HP negative (77.3%). Mean value of the De Meester score was 35,9 ± 56,7 in HP positive patients vs. 33,3 ± 48 in HP negative and this difference was not significant.

Further, there was no statistical difference regarding the severity of symptoms complained by the patients between the two groups (table 4).

**Table 4:** Clinical parameters of 146 GERD patients

**SYMPTOMS**	**HP positive**	**HP negative**	**P**
**Patients**	35/146	111/146	
**Regurgitation (%)**	15 (42.8%)	68 (61.2%)	N.S.
**Dysphagia (%)**	3 (8.6%)	23 (20.7%)	N.S.
**Heartburn (%)**	16 (45.7%)	68 (61.2%)	N.S.
**Epigastric pain (%)**	6 (17.%)	19 (17.1%)	N.S.
**Thoracic pain (%)**	12 (34.3%)	50 (45%)	N.S.
**Dispepsia (%)**	5 (14.3%)	19 (17.1%)	N.S.
**Other symptoms (%)**	6 (17.1%)	23 (20.7%)	N.S.

In addition, to investigate the influence of the above mentioned clinical, endoscopic and functional variables on reflux oesophagitis, a univariate analysis of clinical, endoscopic and functional parameters were performed considering the presence of oesophagitis as independent variable.

We observed that hiatal hernia (p = 0,01), LES opening (p = 0,05), oesophageal wave length (p = 0,01) and pathological reflux number (p = 0,05) were significantly related to the presence of oesophagitis. Differently HP infection was not significantly related to the presence of reflux oesophagitis.

## Discussion

The incidence of HP infection in the patients with GERD, varies widely in literature from 30% to 90% and is approximately of 35% in most series [11].

It was suggested that HP could contribute to GERD through different mechanisms: development of antral gastritis that increases acid production, decrease of LES pressure and impairment of gastric filling [12].

Nevertheless, the decreasing prevalence of HP infection and related diseases (ulcer disease, gastric cancer) in western countries has been paralleled by an increased incidence of gastro-esophageal reflux and related complications. These epidemiological data do not support a causative role of HP for reflux disease, but suggest a negative association [13].

Further, most trials on correlation between HP infection and GERD have indicated no causal relationship [14,15].

Some other authors have even found a lower prevalence of HP infection in patients with reflux symptoms and have suggested a 'protective' role of HP infection against the development of esophageal diseases [16,17]. These authors believe that pre-existing LES dysfunction and gastritis, susceptibility to reflux, increase of a latent reflux are probably causative factors contributing to esophageal diseases rather than HP infection [16].

Patients with HP-related corpus-predominant gastritis may have reduced gastric acid probably mediated by cytokines such as interleukin 1 [13].

Moreover, HP could improve the protective effect of LES by neutralizing acid in the stomach through the activity of urease [18,19]. Furthermore, some authors believe that HP could increase the antisecretory effects of proton pump inhibitors [20-22].

According to Javier and colleagues who found influence of HP infection neither on pH-metric data nor on endoscopic findings [23], in our trial, out of 146 GERD patients, only 24% were HP infected while 76% were HP negative; in addition we found no statistical difference regarding presence and severity of reflux esophagitis between patients with and without HP infection. Besides, Award and colleagues found that HP infection and hiatal hernia in patients with esophageal reflux do not constitute risk factors that affect the severity of esophagitis [24].

Most trials on correlation between HP infection and GERD are based only on endoscopic observations. Actually, endoscopic pattern of GERD patients is often normal; besides, the 24 hours pH-monitoring revealed high diagnostic accuracy for GERD [25,26].

Actually, even if it is undeniable the role of acid secretion in esophageal lesions, it does not seem increased in GERD patients [27-29]. In the present study we found no correlation between HP infection and pH-metric data and the mean value of DeMesteer point was similar in HP positive and negative patients as found by Peters and colleagues [30]. Further, the total time of acidification, was similar in both groups as outlined by Oberg [31], who did not find any correlations between HP infection and esophageal exposure to acid, detected by 24 hours pH-metry, in patients with erosive oesophagitis or Barrett's esophagus.

Schwizer studied 70 patients with GERD treated with lansoprazole associated to clarithromycin and amoxicillin in patients with HP infection. There was no difference in 24-h pH values before and after the HP eradication suggesting that HP eradication did not affect distal oesophageal acid exposure [32].

In addition, we found no significant correlation between HP infection and hiatal hernia, considered by some authors as a supporting element of GERD and significantly associated with the development of oesophagitis [33,34].

Virulent strains of HP, including those with a cytotoxin-associated gene named cagA^+^, have been reported associated to significant gastric inflammation [13]. HP gastritis is accompanied by release of nitric oxide, cytokines and prostaglandins that may impair afferent nerve function, reduce LES pressure and damage esophageal mucosa [35,36]. Differently, according to other authors [36,37] in our trial LES pressure was similar in patients with and without HP infection, further, out of 146 GERD patients only 26% had LES pressure < 14 mmHg, further, LES opening (p = 0,05) and oesophageal wave length (p = 0,01) were significantly related to esophagitis.

Finally, the relationship between HP infection and gastric adenocarcinoma is also controversy. Some authors suggest an increased risk of gastric atrophy in patients HP positive treated with long-term proton pump inhibitor therapy. In a small subset of HP infected patients, chronic gastritis may lead to gastric atrophy and intestinal metaplasia, potential precursor for gastric adenocarcinoma.

In a recent randomized controlled trial by Kuipers, none of the HP-positive GERD patients treated with anti-reflux surgery developed gastric atrophy, compared to 31% of patients treated with proton pump inhibitor therapy for an average of 5 years [38]. Differently, in a long-term trial of GERD patients treated for years with omeprazole, there was an increase both in severity of corpus gastritis and in gastric atrophy in HP-positive patients [39]. Amongst the HP infected patients, atrophy was detected in 12% at baseline and 39% on follow-up.

On the contrary, it has been suggested that HP cagA^+ ^may potentially protect against complications of GERD, such as Barrett's oesophagus and dysplasia/adenocarcinoma [40,41]. The HP infection in patients with Barrett's esophagus has reported in the 12%–60% of patents [33,35,42-46]. A recent meta-analysis presented at Digestive Disease Week 2002 reported a negative association between the prevalence of both H. pylori and cagA^+^H. pylori and reflux disease, Barrett's oesophagus and oesophageal adenocarcinoma [47].

## Conclusion

The exact association between HP and reflux disease continues to be debated. Our clinical, endoscopic manometric and pH-metric data shows significant role of HP infection neither in the development of GERD nor in the pathogenesis of reflux esophagitis. Nevertheless, current data do not provide sufficient evidence to define the relationship between HP and GERD. However, this is an evolving area with ongoing research and further assessments in prospective large studies are warranted.

## Competing interests

The authors declare that they have no competing interests.

## Authors' contributions

MG: manuscript preparation and critical review. FC: literature review and manuscript preparation. MV: manuscript preparation. GMG: data collection and literature review. MGM: manuscript preparation. FR: critical review. AMF: critical review. All authors read and approved the final manuscript.
